# Genome sequence of the lupin-nodulating *Bradyrhizobium sp.* strain WSM1417

**DOI:** 10.4056/sigs.4518260

**Published:** 2013-12-15

**Authors:** Wayne Reeve, Jason Terpolilli, Vanessa Melino, Julie Ardley, Rui Tian, Sofie De Meyer, Ravi Tiwari, Ronald Yates, Graham O’Hara, John Howieson, Mohamed Ninawi, Hazuki Teshima, David Bruce, Chris Detter, Roxanne Tapia, Cliff Han, Chia-Lin Wei, Marcel Huntemann, James Han, I-Min Chen, Konstantinos Mavrommatis, Victor Markowitz, Natalia Ivanova, Galina Ovchinnikova, Ioanna Pagani, Amrita Pati, Lynne Goodwin, Lin Peters, Tanja Woyke, Nikos Kyrpides

**Affiliations:** 1 Centre for Rhizobium Studies, Murdoch University, Western Australia, Australia; 2 Department of Agriculture and Food, Western Australia, Australia; 3 DOE Joint Genome Institute, Walnut Creek, California, USA; 4 Biological Data Management and Technology Center, Lawrence Berkeley National Laboratory, Berkeley, California, USA; 5 Los Alamos National Laboratory, Bioscience Division, Los Alamos, New Mexico, USA

**Keywords:** root-nodule bacteria, nitrogen fixation, rhizobia, *Alphaproteobacteria*

## Abstract

*Bradyrhizobium sp.* strain WSM1417 is an aerobic, motile, Gram-negative, non-spore-forming rod that was isolated from an effective nitrogen (N_2_) fixing root nodule of *Lupinus* sp. collected in Papudo, Chile, in 1995. However, this microsymbiont is a poorly effective N_2_ fixer with the legume host *Lupinus angustifolius* L.; a lupin species of considerable economic importance in both Chile and Australia. The symbiosis formed with *L. angustifolius* produces less than half of the dry matter achieved by the symbioses with commercial inoculant strains such as *Bradyrhizobium sp.* strain WSM471. Therefore, WSM1417 is an important candidate strain with which to investigate the genetics of effective N_2_ fixation in the lupin-bradyrhizobia symbioses. Here we describe the features of *Bradyrhizobium sp.* strain WSM1417, together with genome sequence information and annotation. The 8,048,963 bp high-quality-draft genome is arranged in a single scaffold of 2 contigs, contains 7,695 protein-coding genes and 77 RNA-only encoding genes, and is one of 20 rhizobial genomes sequenced as part of the DOE Joint Genome Institute 2010 Community Sequencing Program.

## Introduction

The *Fabaceae* plant family is the third largest family of flowering plants with a unique ecological role in nitrogen (N_2_) fixation. This family encompasses the three subfamilies *Caesalpinioideae*, *Mimosoideae*, and *Faboideae* (or *Papilionoideae*). The legume genus *Lupinus* (commonly known as lupin) consists of around 280 species classified within the *Genisteae* tribe of the subfamily *Faboideae* with major centers of diversity in South and Western North America, the Andes, the Mediterranean regions, and Africa. This legume has been grown in rotations with cereals for at least 2000 years [1] and is widely distributed within the old and new worlds [2]. The grain may be easily harvested and contains the full range of essential amino acids, and because of its high concentration of sulfur containing amino acids has high feed value for stock [2].

The lupin root nodule bacteria have all been classified within the genus *Bradyrhizobium* [3,4] with the exception of *Microvirga lupini* that was found to nodulate with *Lupinus texensis* [5]. *Bradyrhizobium* spp. are commonly associated with the nodulation of sub-tropical and tropical legumes such as soybean [6,7]. In contrast, lupins are the only agricultural grain legume nodulated by this genus in Mediterranean-type climatic zones. Strains of lupin-nodulating *Bradyrhizobium* are also able to nodulate the herbaceous Mediterranean legume *Ornithopus* (seradella) spp. In this context, lupin *Bradyrhizobium* strains are rare microsymbionts of herbaceous and crop legumes endemic to the cool climatic regions of the world.

The cultivation of lupin in these regions provides a cash crop alternative to soy. *Lupinus angustifolius* in particular has been extensively used to extend grain production into poor quality soils without fertilizer supplementation since fixed nitrogen can be obtained from the symbiosis with *Bradyrhizobium* [8]. Considerable variation exists in the amount of N_2_ fixed in the lupin-*Bradyrhizobium* association [8]. This is significant in agricultural ecosystems, as the benefits derived from growing lupins accrue both to the grain produced and the N_2_ fixed [9]. A well-grown lupin crop may fix up to 300 kg of N per ha. It is therefore important to understand the genetic constraints to optimal N_2_ fixation in this symbiosis. *Bradyrhizobium sp.* strain WSM1417 represents the lower end of the scale in strain N_2_ fixation capacity on *L. angustifolius*, and hence its genome sequence presents an opportunity to understand the genetic elements responsible for this trait. Here we present a summary classification and a set of general features for *Bradyrhizobium sp.* WSM1417 together with the description of the complete genome sequence and its annotation.

## Classification and general features

**Figure 1:**
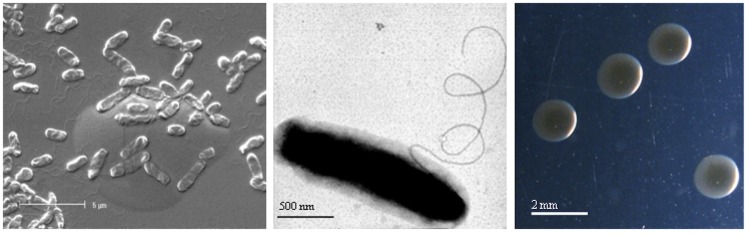
Images of *Bradyrhizobium* sp strain WSM1417 using scanning (Left) and transmission (Center) electron microscopy as well as light microscopy to visualize colony morphology on a solid medium (Right).

**Figure 2:**
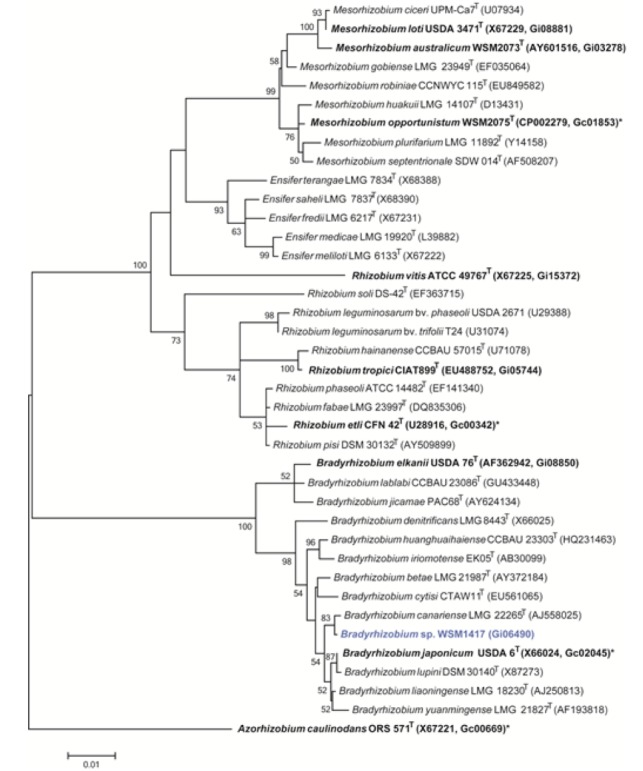
Phylogenetic tree showing the relationships of *Bradyrhizobium sp.* strain WSM1417 (shown in blue print) with some of the root nodule bacteria in the order *Rhizobiales* based on aligned sequences of the 16S rRNA gene (1,334 bp internal region). All sites were informative and there were no gap-containing sites. Phylogenetic analyses were performed using MEGA, version 5.05 [20]. The tree was built using the maximum likelihood method with the General Time Reversible model. Bootstrap analysis [21] with 500 replicates was performed to assess the support of the clusters. Type strains are indicated with a superscript T. Strains with a genome sequencing project registered in GOLD [22] are in bold print and the GOLD ID is mentioned after the accession number. Published genomes are designated with an asterisk.

*Bradyrhizobium sp.* WSM1417 is a motile, Gram-negative, non-spore-forming rod (Figure 1 Left and Center) in the order *Rhizobiales* of the class *Alphaproteobacteria*. It is slow growing in laboratory culture, forming 1-2mm colonies within 7-10 days when grown on half Lupin Agar (½LA) [10] at 28°C. Colonies on ½LA are white-opaque, slightly domed, moderately mucoid with smooth margins (Figure 1C). Minimum Information about the Genome Sequence (MIGS) is provided in Table 1. Figure 2 shows the phylogenetic neighborhood of *Bradyrhizobium sp.* strain WSM1417 in a 16S rRNA sequence based tree. This strain clusters closest to *Bradyrhizobium canariense* LMG 22265^T^ and *Bradyrhizobium japonicum* LMG 6138^T^ with 99.85% and 99.48% sequence identity, respectively.

**Table 1:** Classification and general features of *Bradyrhizobium sp.* strain WSM1417 according to the MIGS recommendations [11,12].

**MIGS ID**	**Property**	**Term**	**Evidence code**
	Current classification	Domain *Bacteria*	TAS [12]
		Phylum *Proteobacteria*	TAS [13]
		Class *Alphaproteobacteria*	TAS [4,14]
		Order *Rhizobiales*	TAS [14,15]
		Family *Bradyrhizobiaceae*	TAS [14,16]
		Genus *Bradyrhizobium*	TAS [17]
		Species *Bradyrhizobium sp.*	IDA
	
	Gram stain	Negative	IDA
	Cell shape	Rod	IDA
	Motility	Motile	IDA
	Sporulation	Non-sporulating	NAS
	Temperature range	Mesophile	NAS
	Optimum temperature	28°C	NAS
	Salinity	Not reported	
MIGS-22	Oxygen requirement	Aerobic	NAS
	Carbon source	Varied	IDA
	Energy source	Chemoorganotroph	NAS
MIGS-6	Habitat	Soil, root nodule, host	IDA
MIGS-15	Biotic relationship	Free living, symbiotic	IDA
MIGS-14	Pathogenicity	Non-pathogenic	NAS
	Biosafety level	1	TAS [18]
	Isolation	Root nodule	IDA
MIGS-4	Geographic location	Papudo, Chile	IDA
MIGS-5	Nodule collection date	1995	IDA
MIGS-4.1	Longitude	-71.452814	IDA
MIGS-4.2	Latitude	-32.521849	IDA
MIGS-4.3	Depth	Not recorded	
MIGS-4.4	Altitude	Not recorded	

Evidence codes – IDA: Inferred from Direct Assay (i.e. first time published); TAS: Traceable Author Statement (i.e., a direct report exists in the literature); NAS: Non-traceable Author Statement (i.e., not directly observed for the living, isolated sample, but based on a generally accepted property for the species, or anecdotal evidence). These evidence codes are from the Gene Ontology project [19].

### Symbiotaxonomy

*Bradyrhizobium sp.* WSM1417 is poorly effective on *L. angustifolius*, producing only 45% of the dry matter compared to that achieved by the commercial inoculant strain *Bradyrhizobium sp.* WSM471 on this species. In contrast on *L. mutabilis*, WSM1417 performs much better, yielding 83% of the dry matter produced by WSM471 on this same host.

## Genome sequencing and annotation information

### Genome project history

This organism was selected for sequencing on the basis of its environmental and agricultural relevance to issues in global carbon cycling, alternative energy production, and biogeochemical importance, and is part of the Community Sequencing Program at the U.S. Department of Energy, Joint Genome Institute (JGI) for projects of relevance to agency missions. The genome project is deposited in the Genomes OnLine Database [22] and an improved-high-quality-draft genome sequence in IMG. Sequencing, finishing and annotation were performed by the JGI. A summary of the project information is shown in Table 2.

**Table 2:** Genome sequencing project information for *Bradyrhizobium sp.* strain WSM1417.

**MIGS ID**	**Property**	**Term**
MIGS-31	Finishing quality	Improved high-quality draft
MIGS-28	Libraries used	Illumina GAii shotgun and paired end 454 libraries
MIGS-29	Sequencing platforms	Illumina GAii and454 GS FLX Titanium technologies
MIGS-31.2	Sequencing coverage	8.1× 454 paired end
MIGS-30	Assemblers	Velvet 1.0.13, Newbler 2.3, phrap 4.24
MIGS-32	Gene calling methods	Prodigal 1.4, GenePRIMP
	GOLD ID	Gi06490
	NCBI project ID	61989
	Database: IMG	2507262055
	Project relevance	Symbiotic N_2_ fixation, agriculture

### Growth conditions and DNA isolation

*Bradyrhizobium sp.* strain WSM1417 was grown to mid logarithmic phase in TY rich medium [23] on a gyratory shaker at 28°C. DNA was isolated from 60 mL of cells using a CTAB (Cetyltrimethylammonium bromide) bacterial genomic DNA isolation method [24].

### Genome sequencing and assembly

The genome of *Bradyrhizobium sp.* strain WSM1417 was sequenced at the Joint Genome Institute (JGI) using a combination of Illumina [25] and 454 technologies [26]. An Illumina GAii shotgun library which generated 82,690,654 reads totaling 6,284.5 Mb, and a paired end 454 library with an average insert size of 10 kb which generated 770,255 reads totaling 144.4 Mb of 454 data were generated for this genome. All general aspects of library construction and sequencing performed at the JGI can be found at the JGI website [24]. The initial draft assembly contained 2 contigs in 1 scaffold. The 454 paired end data was assembled with Newbler, version 2.3. The Newbler consensus sequences were computationally shredded into 2 kb overlapping fake reads (shreds). Illumina sequencing data were assembled with Velvet, version 1.0.13 [27], and the consensus sequences were computationally shredded into 1.5 kb overlapping fake reads (shreds). We integrated the 454 Newbler consensus shreds, the Illumina Velvet consensus shreds and the read pairs in the 454 paired end library using parallel phrap, version SPS - 4.24 (High Performance Software, LLC). The software Consed (Ewing and Green 1998; Ewing et al. 1998; Gordon et al. 1998) was used in the following finishing process. Illumina data was used to correct potential base errors and increase consensus quality using the software Polisher developed at JGI (Alla Lapidus, unpublished). Possible mis-assemblies were corrected using gapResolution (Cliff Han, unpublished), Dupfinisher (Han, 2006), or sequencing cloned bridging PCR fragments with subcloning. Gaps between contigs were closed by editing in Consed, by PCR and by Bubble PCR (J-F Cheng, unpublished) primer walks. A total of 126 additional reactions were necessary to close gaps and to raise the quality of the finished sequence. The estimated genome size is 8.1 Mb and the final assembly is based on 65.8 Mb of 454 draft data, which provides an average 8.1× coverage of the genome.

### Genome annotation

Genes were identified using Prodigal [28] as part of the DOE-JGI Annotation pipeline [29], followed by a round of manual curation using the JGI GenePRIMP pipeline [30]. The predicted CDSs were translated and used to search the National Center for Biotechnology Information (NCBI) non-redundant database, UniProt, TIGRFam, Pfam, PRIAM, KEGG, COG, and InterPro databases. These data sources were combined to assert a product description for each predicted protein. Non-coding genes and miscellaneous features were predicted using tRNAscan-SE [31], RNAMMer [32], Rfam [33], TMHMM [34], and SignalP [35]. Additional gene prediction analyses and functional annotation were performed within the Integrated Microbial Genomes (IMG-ER) platform [24,36].

## Genome properties

**Figure 3:**
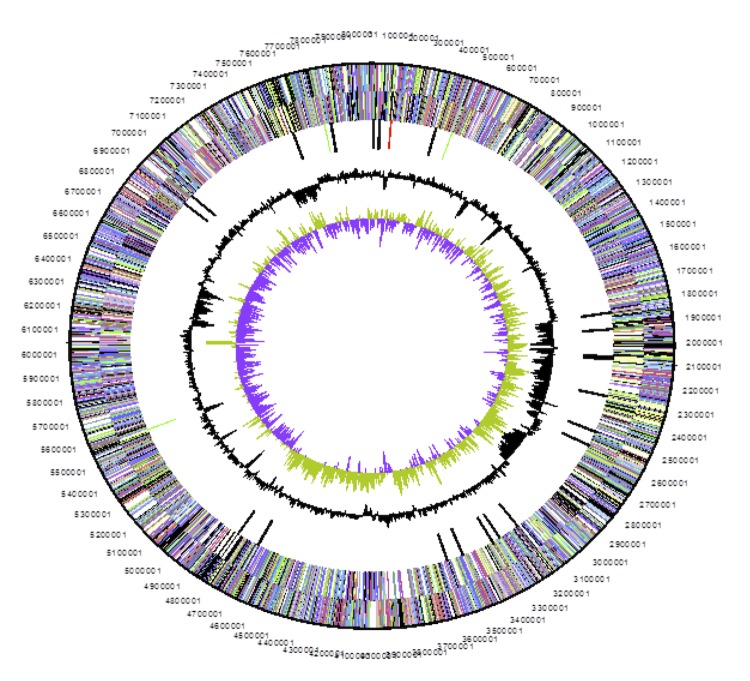
Graphical circular map of the chromosome of *Bradyrhizobium sp.* strain WSM1417. From outside to the center: Genes on forward strand (color by COG categories as denoted by the IMG platform), Genes on reverse strand (color by COG categories), RNA genes (tRNAs green, sRNAs red, other RNAs black), GC content, GC skew.

The genome is 8,048,963 nucleotides with 63.16% GC content (Table 3) and comprised of a single scaffold of two contigs. From a total of 7,772 genes, 7,695were protein encoding and 77 RNA only encoding genes. Within the genome, 272 pseudogenes were also identified. The majority of genes (74.03%) were assigned a putative function whilst the remaining genes were annotated as hypothetical. The distribution of genes into COGs functional categories is presented in Table 4 and Figure 3.

**Table 3:** Genome statistics for *Bradyrhizobium sp.* strain WSM1417.

**Attribute**	**Value**	**% of Total**
Genome size (bp)	8,048,963	100.00
DNA coding region (bp)	6,769,978	84.11
DNA G+C content (bp)	5,084,093	63.16
Number of scaffolds	1	
Number of contigs	2	
Total genes	7,772	100.00
RNA genes	77	0.99
rRNA operons	1	
Protein-coding genes	7,695	99.01
Genes with function prediction	5,754	74.03
Genes assigned to COGs	5,704	73.39
Genes assigned Pfam domains	6,011	77.34
Genes with signal peptides	872	11.22
Genes with transmembrane helices	1,826	23.49
CRISPR repeats	0	

**Table 4:** Number of protein coding genes of *Bradyrhizobium sp.* WSM1417 associated with the general COG functional categories.

**Code**	**Value**	**%age**	**COG Category**
J	202	3.15	Translation, ribosomal structure and biogenesis
A	3	0.05	RNA processing and modification
K	430	6.71	Transcription
L	283	4.42	Replication, recombination and repair
B	2	0.03	Chromatin structure and dynamics
D	37	0.58	Cell cycle control, mitosis and meiosis
Y	0	0.00	Nuclear structure
V	90	1.40	Defense mechanisms
T	354	5.53	Signal transduction mechanisms
M	315	4.92	Cell wall/membrane biogenesis
N	130	2.03	Cell motility
Z	1	0.02	Cytoskeleton
W	0	0.00	Extracellular structures
U	138	2.15	Intracellular trafficking and secretion
O	210	3.28	Posttranslational modification, protein turnover, chaperones
C	417	6.51	Energy production conversion
G	431	6.73	Carbohydrate transport and metabolism
E	678	10.58	Amino acid transport metabolism
F	90	1.40	Nucleotide transport and metabolism
H	235	3.67	Coenzyme transport and metabolism
I	332	5.18	Lipid transport and metabolism
P	331	5.17	Inorganic ion transport and metabolism
Q	244	3.81	Secondary metabolite biosynthesis, transport and catabolism
R	793	12.38	General function prediction only
S	660	10.30	Function unknown
-	2,068	26.61	Not in COGS
